# Malnutrition

**Published:** 1916-11-11

**Authors:** 


### THE SCHOOL DOCTOR.

### Malnutrition.

MALNUTRITION is a fruitful source of inefficiency, disastrous to the nation in peace or war. But its great prevalence among our child population is hardly yet appreciated, nor has its importance as a contributory cause of tuberculosis and other germ diseases received adequate attention. It is the milder degrees of malnutrition which are apt to be overlooked. In a highly civilised country cases of gross starvation are naturally uncommon, but on the other hand several millions of our people earned (before the war) wages which permitted of only 1s. 3d. to 2s. 9d. a week being spent on each child's food. The inevitable result is thousands of children suffering from some degree of proteid starvation or carbohydrate excess; not sufficiently subnormal to be classified in school as "bad nutrition," they come under medical observation later by reason of other diseases due to diminished resistance to germ invasion. Such young persons are the despair of the recruiting sergeant and the employer of labour, or, if girls, the sickly, inefficient mothers of the future.

The part played by actual lack of food in causing malnutrition is far from easy to assess, for the reason that bad *assimilation*, whether due to some systematic disease such as tuberculosis or rickets, or to hygienic errors such as inadequate sleep, has much the same physical effect as an insufficient *intake* of food. In the elementary school population many of these factors may exist in the same child, with the result that the causes of malnutrition are extremely hard to elucidate. It seems clear, however, that among the very severe examples of malnutrition, lack of food is less important than chronic disease, and congenital debility plays a considerable part. Thus among 330 marked cases reported from London schools (London County Council Annual Report, 1914) poverty alone was only found in seventy-seven instances, or 23.3 per cent., whereas tuberculosis was present in seventy-three (22.1 per cent.), other diseases in fifty-seven (17 per cent.), congenital debility (including prematurity) in thirty-four (10.3 per cent.). These conclusions have been confirmed by many other school medical officers, and tend to show that a considerable amount of malnutrition lies outside the remedial scope of the school doctor. But the school can do much by the provision of meals and milk to supplement the deficiencies of home nourishment, and by the teaching of cooking to older girls we have already diminished the amount of under-nutrition.

A serious barrier in the way of effective handling of this admittedly grave problem is the very different meaning attached to the term "malnutrition" by various observers. In the Board of Education Reports it is found that the number of children returned as ill-nourished varies from 34.4 per cent. to 2.7 per cent. in towns of similar character, even where the same method of classification is nominally adopted. This year one great Northern town boasts not a single badly nourished child, and only .81 per cent. even "below the average." Obviously, a scientific formula for estimating nutrition is badly wanted, and numerous efforts have been made to construct one, but without much success. It was hoped that the ratio between height and weight (or more accurately the nutritional index

$$\frac{100\sqrt[3]{\text{weight in kilograms}}}{\text{height in centimetres)}}$$

would furnish a convenient and reliable standard, but after much investigation it has been found that the results are distorted by seasonal variations in growth, and vary irregularly with age. It is only suitable for comparing large groups of children, and not for dealing with small changes in small groups or individuals. The Oppenheimer Index (arm circumference × 100÷chest circumference), though much lauded by German school hygienists, has proved unsatisfactory in practice.

For practical purposes nothing at present devised can replace the personal estimate of a trained observer. But this empirical standard can only give results of real value if certain precautions are observed. Firstly, care should be taken by the examiner to preserve a fixed mental picture of a really well-nourished child, "of good social position," as Dr. Mackenzie of Dunfermline wisely suggests. The obvious tendency is to shift the standard and to take as one's average or normal child the average of the group under observation for the moment. This entirely vitiates results for poor schools. Secondly, the protean character of the signs of malnutrition might well be better recognised. Too much emphasis is laid on the presence or absence of subcutaneous fat, while the significance of harsh, inelastic skin, dull, lustreless hair and eye, anæmia, muscular flabbiness, lassitude, nervous disturbances, are apt to be overlooked. Only thus can the grave discrepancies due to the "personal equation" be diminished and the whole problem put on a more scientific basis.

